# Histomorphometric changes in testis following administration of tenofovir nanoparticles in an animal model

**DOI:** 10.1186/s11671-024-04002-y

**Published:** 2024-03-25

**Authors:** Edwin Coleridge Naidu, Samuel Oluwaseun Olojede, Sodiq Kolawole Lawal, Onyemaechi Okpara Azu

**Affiliations:** 1 Discipline of Clinical Anatomy, Nelson R. Mandela School of Medicine, University of KwaZulu-Natal, Durban, South Africa; 2 Department of Human Biology, Faculty of Health Sciences, Walter Sisulu University, Mthatha, 5117 South Africa; 3 School of Nursing Sciences, Faculty of Health Sciences, University of Botswana, Gaborone, Botswana; 4 Department of Medical Biosciences, University of the Western Cape, Bellville, South Africa

**Keywords:** Tenofovir nanoparticles, Testis, Ultrastructure, Human immunodeficiency virus

## Abstract

**Background:**

Nanoparticle-based drugs are new inventions in the management of the Human immunodeficiency virus (HIV) pandemic, especially resistant forms of the virus in anatomical sanctuary sites and organs such as the testis. However, safety issues must be resolved to attain the optimal potential of newer nano-drug formulations.

**Aim:**

The study investigated the toxicological potential of synthesized Tenofovir Nanoparticles (TDF-N) on testicular indices when used for the prevention and treatment of HIV.

**Methodology:**

Fifteen male Sprague–Dawley (SD) rats with weight ranging from 230 g to 250 g were randomly assigned into groups A (control, saline), B (TDF), and C (TDF-N). The testes were removed for sperm analysis and processed for H/E and PAS stains. Cell counts and cellular measurements; the diameter and the area of the testicular seminiferous tubules were measured using ImageJ and Leica software 2.0.

**Results:**

A significant reduction (*p* < 0.05) in sperm count was noticed in the TDF-N group. Also observed in the TDF and TDF-N groups was a significant reduction (*p* < 0.05) in sperm motility and in the number of dead sperms compared with the control. Sperm abnormalities such as distorted basement membranes, loss of germ cells, hypocellular interstitium, and loss of spermatogenic series were increased in the TDF and TDF-N groups. There was also a significant reduction (*p* < 0.05) in the cell count, diameter, and area of seminiferous tubules observed in these groups.

**Conclusion:**

TDF and TDF-N may be detrimental to the testis and testicular tissue, leading to significantly reduced sperm counts, motility, and ultimately–male fertility.

## Introduction

The development of nanoparticles (NPs) with unique physicochemical properties has revolutionized healthcare treatment, allowing for greater and more efficient use of therapies hitherto problematic due to issues of penetration or accessibility to target tissues [1]. This has been largely possible because of the particles very high surface area-to-volume ratios with its consequent ability to greatly modify their biophysical and biochemical characteristics. Nanomaterial use in medicine could be in the form of drug delivery systems, gene therapy [2, 3], fluorescent biological labels, imaging contrast [4], or microsurgical technology [5]. Nanomedicine has provided a new and powerful tool for tissue regeneration, imaging, and in the development of new medical devices [1, 6, 7].

Nanocarriers to improve drug delivery in HIV infections have evolved with tenofovir (TDF) nanoemulsion systems employing novel dendritic ester derivatives (NDED) with proven superior transdermal penetration [8]. This is a useful tool with the capacity to enter HIV reservoirs within the testes which are still reported to persist despite treatment with highly active antiretroviral therapy (HAART) [9]. This phenomenon may be linked to a potential inadequacy of antiretroviral drugs (ARVs) to enter into the seminal fluid of HIV-positive men undergoing treatment in therapeutic concentrations [9–16].

Whilst extensive research support for nanotechnology-driven innovations continues to generate wide attention in the Republic of South Africa (as seen globally) [17], especially within the health sector, concerns relating to safety [18] remain challenging. Some experts express concerns about how the interactions between the constituent elements of nanoparticles and the biological/tissue samples could lead to deleterious effects. There is a pressing need to fully comprehend the cellular responses that occur when nanomaterials undergo biological degradation inside the cell. These particles may build up inside the cellular environment and result in intracellular changes, which include gene distortion and alteration of organelle integrity. The hepatotoxicity and nephrotoxicity of Zirconia oxide nanoparticles in experimental rats have been documented [19]. In vivo studies have reported increased allergic vulnerability in the offspring of mouse dams that were intranasally insufflated with NPs [20]. Other adverse effects were reported on spermatogenesis, morphological alterations in testicles, and gene expression in the brain following maternal subcutaneous injections of TiO_2_ nanoparticles [20].

Growing body of evidence implicating nano-delivery particles needs urgent attention as there is increasing interest in the delivery of nanoformulated antiretroviral drugs to overcome the adverse complications. Notably, recent in-vivo and in-vitro studies have demonstrated advances and promising results have been noted when TDF-loaded nanoparticles were studied on various cell lines and rat organs for their potential application to manage HIV and associated complications [21–23]. Although, the current HIV treatment regimen in South Africa containing the preferred first-line ART combination of tenofovir disoproxil fumarate, lamivudine, and dolutegravir (TLD) has produced better treatment outcomes such as viral load suppression, enhanced immune response, and improved clinical indices [24–26]. Emerging studies have documented several adverse effects including virologic failure and cardio-metabolic disorders secondary to excessive weight gain following initiation and treatement with dolutegravir-based regimen [27–29]. The dearth of literature on the potential toxicity of Tenofovir Nano- antiretroviral delivery systems poses enormous challenges to the health of patients and the outcome of therapeutic interventions. Thus, this study therefore was designed to fill the gap in knowledge on the effects of TDF-N on the testis of experimental rats.

## Materials and methods

### Ethical statement

This study was performed following the ARRIVE guidelines and the National Council’s guide for the care and use of laboratory Animals.

### Ethical clearance

Ethical approval for this study was received from the Animal Ethics Committee of the University of KwaZulu-Natal (UKZN), South Africa with approval number AREC/010/016PD.

### Animal care

A total of fifteen male Sprague-Dawley (SD) rats, kept under standard animal house conditions at the Biomedical Resources Unit, UKZN, were used for this study. The rats received humane care following the guidelines of the National Council for Care and Use of Laboratory Animals. Various environmental enrichments such as play tunnels and nutritional supplements like sunflower seed were offered to the Animals to cater to their psychological welfare in such a way that emulates the natural environment of captive animals and prevents abnormal behaviour. The baseline body weights were measured before the rats were randomly assigned into three (3) groups of five (5) rats each.

### Experimental design

Fifteen adult male SD rats weighing between 230 and 250 g were randomly assigned into 3 groups viz- Group A control which received normal Saline, Group B was administered with TDF (4.3 mg/kg), Group C was administered with TDF-N (4.3 mg/kg). All animals were injected intraperitoneally once daily for 4 weeks between 8:00 am and 10:00 am and this time was kept constant throughout the experiment.

#### Preparation of nanoparticles

Tenofovir disoproxil fumarate (TDF) was bought from Sinobright Pharmaceutical Company Limited (China). Also, triethylamine, Solutol HS 15®, hydroxypropylmethylcellulose (HPMC), and PEG 400, were procured from Sigma-Aldrich Co.Ltd., (USA). Milli-Q water purification system (Millipore Corp., USA) was used to obtain purified water to prepare formulations. TDF-N was synthesized following the procedure described by Rambharose et al. [30]. The nanoemulsion was produced using an ultra-sonication technique by mixing the S_mix_ and oily phase Linolenic acid and adequate milli-Q water. The blank nanoemulsion was formed by sonicating (Ultrasonic Homogenizer, Kennesaw, USA) the solution at 20 °C for 10 minutes at an amplitude of 30%. Thereafter, the Tenofovir-loaded nanoemulsion was prepared by the emulsification process in a temperature-regulated ice bath [30]. Drugs were dissolved and prepared for administration at calculated human therapeutic dose level equivalentsof 4.3mg/kg body weight.

#### Weight determination

The body weight of the rats was taken by electronic weighing balance (Zeiss West Germany (Pty) Ltd) on the first day of the experiment, and consequently weekly at the same times each day, while the last weight was taken on the last day of the experiment.

#### Animal sacrifice and collection of samples

Twenty-four (24) hours after the last administration, the rats were euthanized by excess Halothane after which blood samples were collected via transcardial puncture. From each rat, five milliliters (5 ml) of blood were obtained into a plain bottle and the serum obtained by centrifuging at 3000 rpm for 10 min. The testes were removed and separated from the cauda epididymis. The testes (both left and right) were weighed individually by electronic balance (Mettler Toledo; Microstep (Pty) Ltd., Greifensee Switzerland), and the average for each animal was recorded. One testis per rat was fixed in Bouin`s fluid for histological analysis.

#### Sperm motility, sperm count, and sperm morphology

The cauda epididymis from each rat was minced in 5 milliliters of normal saline and used for the analyses of sperm morphology, sperm motility, and sperm count. The sperm motility examination was performed by preparing a drop of epididymal fluid on a glass slide, covering it with a coverslip (22 × 22 mm), and examined under a light microscope [31]. The microscope fields were scanned, and sperm motility was assessed and graded as dead, progressive, and non-progressive. A minimum of, 10 microscope fields were examined at 400× magnification. The relative percentage of motile spermatozoa of all examined fields was documented to the nearest 5% using a consistent subjective method [31, 32].

The standard hemocytometer method was employed for the epididymal spermatozoa counts. The epididymal fluid was carefully minced and 10 μl of this diluted specimen was transferred to each side of the counting chamber on the hemocytometer. Thereafter, the sperm counts were counted with Bio-Rad equipment and the average was computed to the nearest millions /milliliter [33].

The spermatozoa morphology was examined using dry-smeared spermatozoa stained with eosin-nigrosine placed on a glass slide and observed under a light microscope (Leica DM 500) at 400× magnification. The percentage of normal sperms, and sperms exhibiting abnormal heads, abnormal tails, and abnormal midpieces were documented [34].

#### Relative organ weight

The percentage relative organ (testicular) weights were determined by using the formula stated below [35, 36]:

$${\text{ROW}}=\frac{Organ \,weight}{\mathrm{Total\, body\, weight }}X 100$$

### Histomorphometric studies

Bouin’s fluid was used to fix the testicular tissue and then transferred to 70% ethanol. These tissues were subsequently processed by graded ethanol sets, embedded in paraffin and sectioned into 5 µm-thick slices using a microtome (microm HM 315 microtome, Walldorf, Germany). Thereafter, the sections were stained with hematoxylin and eosin (H&E) and periodic acid Schiff (PAS) and subsequently scanned and captured by the Leica Microsystems.

To perform the histomorphometric examination, approximately seven sections on a vertical axis starting from the polar to the equator were studied using unbiased stereological techniques to measure the seminiferous tubular area and height [37].

The systematic random sampling technique was adopted to examine the seminiferous cell count to ensure fair distribution from the sampled sections. In total, eighteen seminiferous tubules exhibiting round profiles were randomly picked from each slide. The vertical diameter of the seminiferous tubule was denoted as d1, and the horizontal diameter was indicated as d2 while the mean diameter indicated as (D) was documented as an observation. This was employed to reduce the longitudinal profiles that showed different degrees of irregular shrinkage or damage [37]. The diameter and height of the seminiferous tubular epithelium were scanned using Leica SCN 400 (Leica Microsystems GmbH, Wetzlar, Germany) and measured using the image analyzer and Leica microsystem software, while cell counts were done through image J software. To compute the cross-sectional area of the seminiferous tubule, the formula given below was employed [33];

$$Area=\frac{\mathrm{\pi D}2}{4}.$$

where ***π*** equal to 3.142 and D is the mean of the diameter of the seminiferous tubule.

### Statistical analysis

The data obtained were subjected to statistical analysis using a one-way ANOVA followed by Fisher’s least significant difference (*post-hoc)* test using Statistical Graph Pad version 6.0. For the results obtained, *P* < 0.05 was regarded as statistically significant.

## Results

### Weight difference

**Fig. 1:**
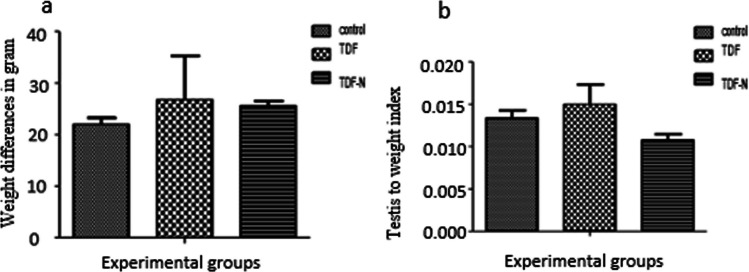
This figure shows the weight changes **a** and relative testicular weight **b** among the treated and control groups. The Y-axis in **a** represents the weight difference in grams while the X-axis denotes the experimental groups (Control, TDF, and TDF-N). Also, the Y-axis in **b** indicates the testicular weight index while the X-axis denotes the experimental groups (Control, TDF, and TDF-N)

There were no weight differences between the control and treated groups (Fig. 1a). Also, we reported no significant difference (*p* < 0.05) in the relative testicular weight between the Nano groups and control (Fig. 1b).

### Sperm parameter

**Fig. 2:**
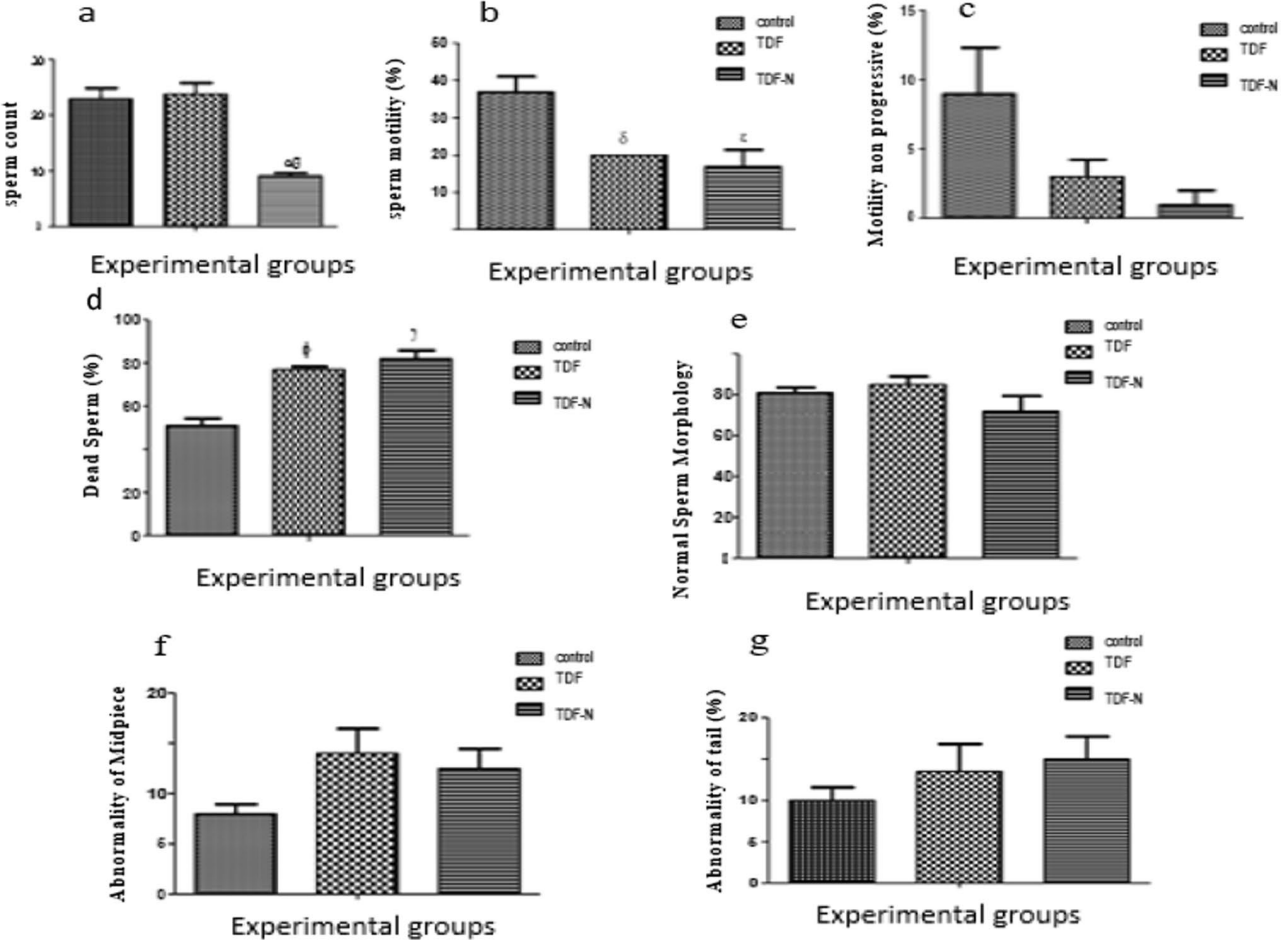
These figures show the normal and abnormalities of Sperm morphology (**a**), Sperm count (**b**), sperm motility progressive (**c**), motility non-progressive (**d**) dead sperm (**e**) normal sperm (**f**) abnormality of midpiece, (**g**) abnormality of tailpiece. The Y-axis represents the percentage/concentration/count while the X-axis denotes the experimental groups (Control, TDF, and TDF-N)

A significant reduction (*p* < 0.05) in Spermatozoa count was observed in the TDF-N group when compared with the control (Fig. 2a). Sperm motility was significantly reduced (*p* < 0.05) in both TDF-N and TDF-treated animals (Fig. 2b and c). Also, a significant increase (*p* < 0.05) in dead sperm count was observed in the treated groups (B and C). Sperm morphology revealed no significant difference (*p* < 0.05) across the groups (Fig. 2d and e). There was, however, a non-significant increase in abnormality of the midpiece (Fig. 2f) and tail (Fig. 2g) in the TDF and TDF-N groups. In contrast, no abnormalities were observed in the head in both the control and treatment groups (Fig. 2f).

### Seminiferous tubular diameter and area count

**Fig. 3:**
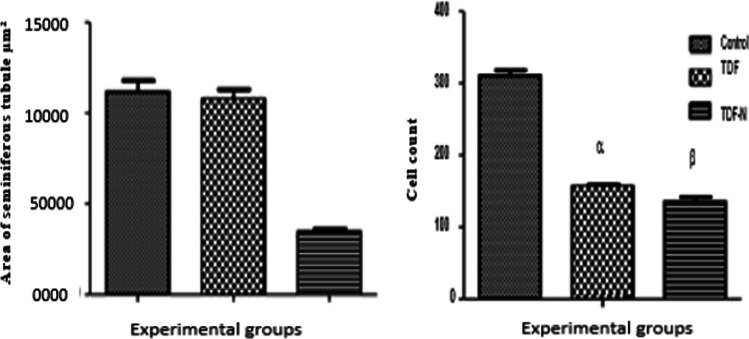
This figure depicts the stereology of the seminiferous tubular area and cell count in treated and control animals. There was a decrease in the area of the seminiferous tubule in the TDF-N (β) group compared to TDF (α) and control. A significant decrease in cell count was also observed in both TDF and TDF-N groups compared to control. The statistical significance difference was performed by One-way ANOVA followed by Fisher’s exact test, where TDF-N (^β^p < 0.05), vs. Control and TDF; Also, TDF & TDF-N (^αβ^p < 0.05) vs. control. The Y-axis in **a** represents the area of the seminiferous tubule in µm^2^ while the X-axis denotes the experimental groups (Control, TDF, and TDF-N). Also, the Y-axis in **b** indicates the cell count while the X-axis denotes the experimental groups (Control, TDF, and TDF-N)

In this study, a decrease in the diameter and area of seminiferous tubules among TDF-N animals was observed compared to control and TDF animals (Fig. 3a). In addition, a decrease in cell counts among TDF and TDF –N animals was noticed compared to controls (Fig. 3b).

#### Histology of the testis

**Fig. 4:**
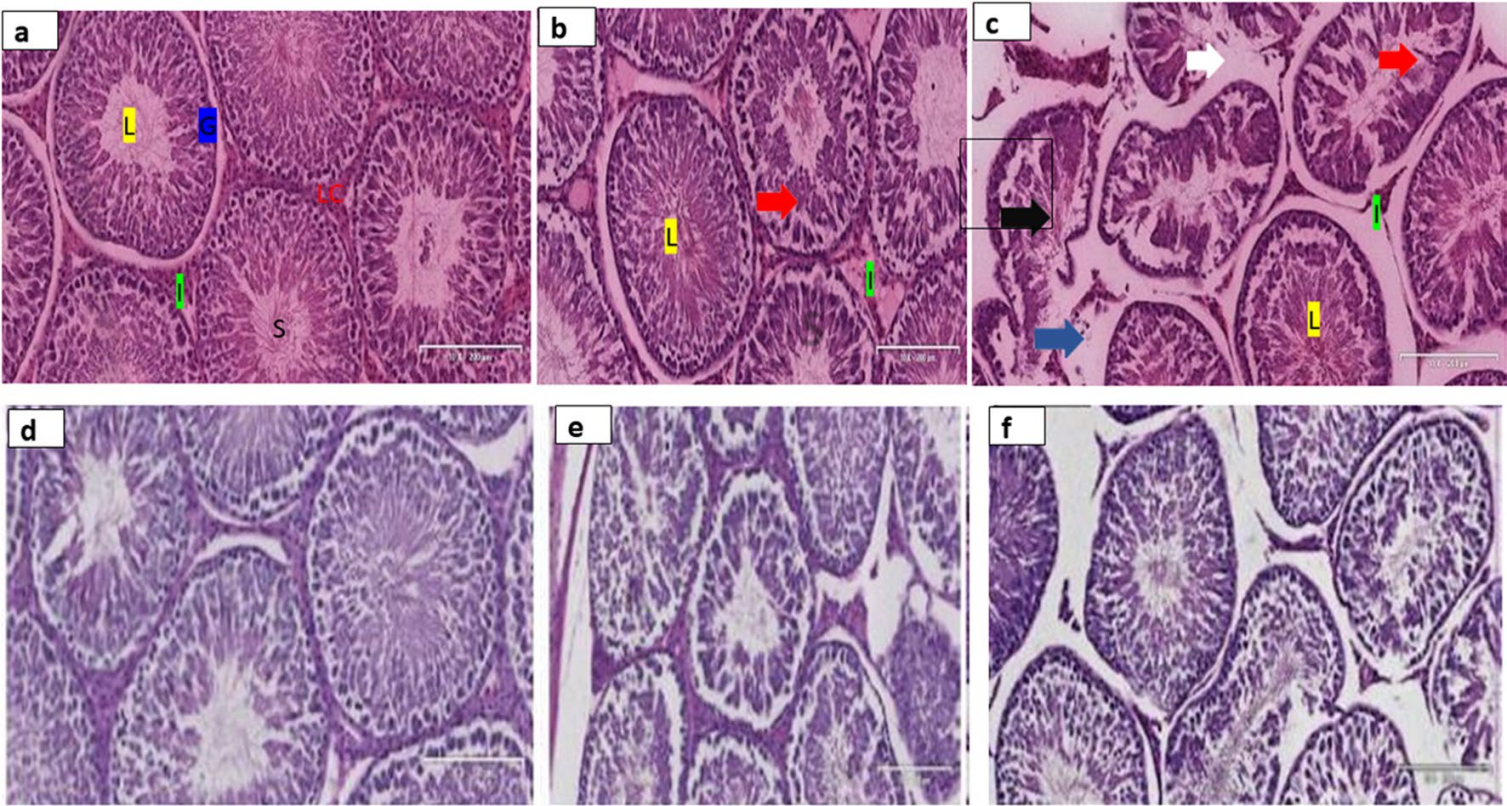
These photomicrographs show the H&E staining, Control (**a**), TDF (**b**), TDF-N (**c**) as well as PAS staining, Control (**d**), TDF (**e**) and TDF-N (**f**). Testicular sections of control animals stained with H&E (**a**–**c**) showed well-preserved cellular orientation in germinal cell epithelium (G), normal interstitial spaces (I) with well- populated Leydig cells (LC), and normal lumen (L) with spermatozoa (S). In contrast, the histology of animals in TDF and TDF-N (H &E) displayed loss of germ cells (red arrow), alteration of basement membranes (white arrow), widened interstitium (blue arrow), and loss of spermatogenic lineage with shrunken seminiferous tubules (black arrow). In addition, increased staining intensity (PAS) was observed in TDF and TDF-N groups compared to control (**d**–**f**)

The testicular section of the control animal stained with H & E (Fig. 4a–c) showed a well-preserved cellular orientation in the germinal cell epithelium (G), normal interstitial (I) space with well-populated Leydig cells and normal lumen filled with immotile spermatozoa (S). In contrast, the histology of animals in TDF and TDF-N (H & E) displayed a loss of germ cells, widened interstitium, altered basement membrane, and loss of spermatogenic lineage with shrunken seminiferous tubules. In addition, increased staining intensity (PAS) was observed in TDF and TDF-N groups compared to control (Fig. 4d–f).

## Discussion

Limitations associated with current available HIV drugs contribute significantly to poor therapeutic outcomes and suboptimal patient management. Conventional drugs have limitations in crossing the blood testes barrier to attack HIV. Nanoformulated-drugs have great potential to attack HIV sequestered in sanctuary sites [38]. This potential may not be realized unless safety concerns are met. This study was carried out to investigate the effects of TDF-N on the testis of Sprague Dawley rats using standard laboratory procedures.

It was observed that chronic exposure to TDF-N and TDF for 30 days did not induce any abnormality in weights or relative testicular weight when compared with controls. This suggests that the drug did not interfere with the feeding habits of the laboratory animals. In a similar study, Peter and colleagues [39] observed no significant effects in the weights and relative organ weights among TDF and TDF-loaded nanoparticles. Similarly, this report agrees with Ng. Stock [40] who found no significant effects on the body weight of the experimental mice following 13 weeks of oral administration of TDF.

Also observed are significant perturbations in sperm parameters with a significant reduction in sperm count in the TDF-N group when compared with the control group, indicating that the TDF-N was toxic to sperm cells. This result agrees with a previous study which revealed that nanoparticles accumulate in organs such as the testis, destroying the Sertoli, Leydig, and germ cells to alter sperm indices [41]. The exact mechanism of toxicity of nanoparticles however still remains unclear. Studies have suggested that nanoparticles produce reactive oxygen species (ROS), which alters the concentration of intracellular calcium, activates transcription factors, and modifies the inflammatory biomarkers [42, 43]. Although, recent finding shows that the activity of ROS is required for spermatozoa to attain morphological and functional maturity, they are nevertheless more vulnerable to oxidative stress damage than any other type of cell [44].

The observed reduction in sperm motility and the significant increase in the number of dead sperms in the TDF group indicate toxic effects of TDF. Similar clinical cohort and in vivo studies have documented a deleterious effect of TDF on semen and sperm indices such as count, motility, and morphology [45–47]. Previous studies have implicated highly active antiretroviral therapy in various reproductive perturbations such as a significant decrease in sperm count, alteration to spermatozoa, and increased abnormal sperm forms [48, 49]. All these findings taken together, indicate the adverse effect of TDF on sperm indices and morphology.

Moreover, sperm morphology including normal, head, mid-pierce, and tail abnormalities was not significantly different amongst the treated groups, suggesting that a short duration of exposure (4 weeks) to TDF and TDF-N might be responsible for this negative finding. This is on account that many factors including long-term exposure to antiretroviral therapy and nanoparticles have been described as major causes of organ toxicity [50, 51].

There is a clear indication that TDF-N reduces both sperm count and motility and as such could cause male infertility which accounts for approximately 50% of overall (primary and secondary) infertility. It is important to note that sperm analysis provides an initial evaluation and useful information concerning sperm count, sperm motility and viability, and patency of the male genital tract, however, it is not a definitive test of fertility [52].

The observed reduction in area and alterations in the seminiferous tubules in both the TDF-N and TDF groups suggests testicular injury which corroborates a previous study that linked alterations on seminiferous tubules such as epithelial sloughing and shrinkage to testicular perturbations [53]. In addition, the observed increase in collagen deposition in the TDF-N group strongly suggests testicular inflammation. Collagen is a powerful marker of fibrosis in testicular tissues, and its increase usually occurs following tissue inflammation and cellular injury [54]. Nanoparticles concentrate in testicular tissues [55] and may account for the changes reported in this study.

In summary, the conjugation of tenofovir disoproxil fumarate with nanoparticles (TDF-N) represents an innovative approach in antiretroviral therapy, particularly aimed at enhancing drug delivery and efficacy against HIV. This combination aims to leverage the unique properties of nanoemulsions which improve drug distribution, targeted delivery, and enhance antiviral activity while also potentially reducing toxicity and resistance [56].

## Conclusion

Various stakeholders have acknowledged that potential health, environmental, and safety risks of nanomaterials may hinder nanotechnology from reaching its full potential. In this study, it has been shown that TDF-N may predispose to degenerative changes of the seminiferous tubules with disruption of the spermatogenic series, abnormality of sperm parameters, and testicular fibrosis. Hence, since sperm count and motility are critical components of sperm analysis, patients on this drug (TDF), as well as nanodrug (TDF-N), should be cautioned on the reproductive consequences.

### Limitations of the study

Although this study provides some insight into the status of testicular histomorphology following the administration of TDF and TDF-N in a rat model, further studies should investigate the effect of TDF-N on the testicular antioxidant defense system, male reproductive hormones, and testicular modulators that were not reported in this study.

## Data Availability

All the data generated during research and analysis to support the findings of this research are embedded in this manuscript.

## Abbreviations

ANOVA: Analysis of variance
ARVs: Antiretroviral drugs
H&E: Hematoxylin and Eosin
HAART: Highly active antiretroviral therapy
HIV: Human Immunodeficiency Virus
NDED: Novel dendritic ester derivative
NPS: Nanoparticles
PAS: Periodic acid–Schiff
ROW: Relative organ weight
SD: Sprague–Dawley
TDF: Tenofovir Disoproxil fumarate
TDF-N: Tenofovir Nanoparticle
